# Proceedings of Terrell Medical Society

**Published:** 1888-02

**Authors:** L. M. Stroud, W. H. Monday

**Affiliations:** Forney, President; Terrell, Vice-President


### PROCEEDINGS OF THE TERRELL MEDICAL SOCIETY.

*Dr. L. M. Stroud, of Forney, President, Dr. W. H. Monday, of Terrell, Vice-President.*

For Daniel's Texas Medical Journal.

Dr. W. P. Dumas, of Elmo, read an able paper upon the subject of Antisepsis.

Dr. G. A. Nelson cited a case of abdominal section which made an excellent recovery under antiseptic treatment, and thought that it should never be omitted, as the chances for a good recovery of the patient were considerably augmented thereby.

Dr. John Inabnit cited a case in which the greater portion of the intestines were exposed and covered with dirt, which were well washed and returned without the use of antiseptics, and thought the free use of cold water could be depended upon, without the use of antiseptics, although he recognized their value.

Dr. W. T. Strain reported a case of mammary abscess, which being freely laid open, to which he applied iodoform unsparingly; the patient made a good recovery.

Dr. Stroud propounded the question, why can we not destroy in the blood the germ which produces puerperal fever, and thus obviate the great loss of life caused by this factor? and thought that if bi-chlor. of mercury effectually destroyed the germ topically, it might act in antagonism when administered internally.

Dr. Stroud cited a case of amputation of a toe, in which case he used an ointment composed of cocaine, carbolic acid and lard, which prevented suppuration, and which healed by immediate union.

Dr. Nelson indorsed this mode, having made use of a similar preparation.

Dr. White stated that this ointment was a fine application is cases of hemorrhoids.

Dr. Nelson stated that his experience taught him that, as a rule, we would find suppurating buboes as a result of glandular contamination.

Dr. White had no recollection of having met with such cases.

Dr. W. G. Hardin having withdrawn from his compact with Stovall & Pollard, applied in a written application for reinstatement as a member into our Society, which was favorably received, and having complied with the By-Laws and Constitution of this Society, his name was placed upon its roll of members.

Dr. Stroud cited a case of erysipelas attacking the entire surface of a child.

Dr. Inabnit said that he applied a strong solution of argenti nitras sufficiently to blister the parts to which it is applied.

Dr. Garrett, of Forney, said he had used acidum tannicum, glycerina, ferri et quinia successfully

Dr. Anthony said his most satisfactory treatment was belladonna and glycerine externally.

Dr. Dumas said that he was partial to the use of Monsels' Solution of Iron, with glycerine internally, and argenti nitras solution externally.

Dr. Smiley said his preferred remedy was phytolacca fll. extract externally, and *quinia et ferri* internally.

Dr. Nelson said that he would be very loth to make any application which would leave a shield-like covering, and would elect tannic acid and sherry wine, and large doses of iron internally. He spoke of the treatment of diphtheria, and stated his confidence in the use of the steam atomizer, using as a spray, carbolic acid, cocaine and glycerine.

Dr. Inabnit did not hold to the contagiousness of diphtheria, while every other member of the Society held otherwise.

Dr. Smiley read a paper upon incontinence in children, and stated that Belladonna had proved a very efficient treatment, and in more obstinate cases he had introduced a bougie immersed in a 10 per cent solution of argenti nitras, letting it remain from one to two minutes once a week.

Dr. Inabnit said that he was partial to the use of cantharides and ergotin.

Dr. Yates had used, with fine results, balsam copaiba and belladonna.

Dr. Garrett uses ergotin, belladonna and nux vomica, following with *potassii bromidum*.

Dr. Nelson stated that as there is always an abnormally acid condition of the urine, it should be examined, as the first step in the treatment, and favored ergotin as an efficient remedy.

The election of officers for the ensuing term resulted as follows:

Dr. L. M. Stroud, of Forney, as President.

Dr. W. H. Monday, of Terrell, as Vice-President.

Dr. E. E. Smiley was re-elected Secretary and Treasurer.

E. E. SMILEY,
Sec. and Treas.

---

THE TRAVIS COUNTY MEDICAL SOCIETY held its regular monthly meeting in Austin Thursday, February 2, 1888, Dr. A. N. Denton. President, in the chair. There was an unusually large attendance, and much interest manifested. Dr. McLaughlin, who had been appointed to present the subject for discussion—Dysentery, read a paper in which the subject was outlined, and the treatment reviewed. Dr. Wooten opened the discussion, and gave a resume of his extended experience in the treatment of the formidable malady. He was followed by Drs. Q. C. Smith, R. M. Swearingen, J. M. Litten, T. J. Bennett, J. F. Hamilton, W. A. Morris, B. E. Hadra, and other gentlemen present. The paper, with a synopsis of the discussion, will appear in the March number of the JOURNAL.

---

THE PAPER of Dr. Q. C. Smith on Acute Pulmonic Inflammations in children, which appears in this issue, was read before the Travis County Medical Society at a late meeting, by appointment, and was discussed by Drs. Swearingen, Tyner, Bennett, Daniel, Dean, and Dr. Carhart, of Lampasas, a visiting brother. It was intended to embody their remarks in a report of the meeting, to be published with the paper, but the secretary failed to supply the manuscript in time.

---

T. S. M. A. SECTION ON GYNÆCOLOGY; Dr. Christian requests us to insert the following:

" The profession are respectfully invited to contribute to the Gynæcological Section of the Texas S. Med. Association at the April meeting. All papers should be sent to Dr. John C. Jones, Secretary, Gonzales, Texas, by April 10.

Respectfully,

G. W. CHRISTIAN, Chm.

